# Sexuality and other issues in Africa and beyond

**DOI:** 10.4314/ahs.v18i2.1

**Published:** 2018-06

**Authors:** James K Tumwine

**Affiliations:** EIC, African Health Sciences

Welcome to this June 2018 issue of *African Health Sciences*. It focusses on sexuality, non-communicable diseases, HIV, child health and other challenges besetting the health systems in low and middle income countries in Africa and other environs.

*Now to sexuality:* Researchers from Nigeria report on the burden and pattern of child sexual abuse among adolescents. They found a 25% prevalence among adolescents studied. Most perpetrators were boyfriends or neighbours, and only a third of the cases reported the abuse.^1^

Kenyan researchers on the other hand^2^ report on sexual experiences of 200 secondary school students in Nakuru, where a large number had had sexual experiences. The work by Adimora^3^ and others, on the other hand, brings to the fore the predictive power of the home environment and peer pressure on disruptive and risky sexual behaviour of adolescents.

Now to sexuality of pregnancy: Iranian authors assessed effects of pregnancy on sexual function of couples. They report that sexual dysfunction is a widespread problem during pregnancy among Iranian couples. This paper makes very interesting reading and it is highly recommended.^4^

Togolese scientists studied the effect of leaves of *S.mombin* on uterine muscle contraction in child birth.^5^ They concluded that the hydro-ethanolic extract of *S.mombin* leaves exert their effect through prostaglandins release, α2-adrenoreceptors stimulation and calcium release, and subsequent uterine smooth muscle contraction.

The next paper^6^ by Kumuda and others reports on salivary cortisol levels in postmenopausal women with psychosomatic disorders. They found that salivary cortisol was higher in post-menopausal women with clinically diagnosed psychosomatic disorders, than in those without. To conclude this sexuality section, we bring you the seminal work by Stella Gbotoloru who found that quinine blocks ovulation and induces oxidative stress in the ovary.^7^

## Non Communicable Diseases

In the next section, we bring you very interesting papers on non-communicable diseases. Shen and others have developed a cytokine signature from peripheral blood that serves as a fingerprint for hepatocellular carcinoma diagnosis. 8 Chinese authors have demonstrated that ‘572G/C polymorphism of the IL-6 gene may be a risk factor for the development of prostate cancer in Asians.’^9^

On the other hand, a study from South Africa casts doubt on the ‘yield of interpretable test result of fine needle aspiration cytology when compared to breast ultrasonography’, prompting them to recommend omission of FNAC from the triple assessment of women with suspected breast cancer.^10^ The work by Jibrin and others^11^ highlights the histo-pathological pattern of brain tumours in Nigeria. The rest of the NCD papers, deal with diagnosis of ischemic disease^12^, rheumatoid arthritis^13^, the retina^14^, hearing loss^15^, limb injury^16^, poisoning^17^, and glaucoma.^18^

## Child Health

We have a few papers on child health. South African clinicians bring us an interesting paper on congenital abnormalities found during circumcision.^19^ Oral mutilation of infants has been reported in East Africa, but no substantial work had been reported from the Sudan. We bring you an interesting treatise on this subject by Elgamri and others.^20^ They found that 10% of the children studied had infant oral mutilation and identified several associated factors.

The other papers are on neonatal mortality in Ghana^21^, and new born thyroid function.^22^

## Infections

HIV of course is the big story here, with authors reporting adherence to ART^23^, physical activity^24^ and disputes over HIV diagnosis by ELISA^25^, hepatitis C^26^ and surveillance of epidemic prone diseases.^27^

Finally, we bring you papers on uroliths^28^, kidney disease^29^, childhood cervical spine injuries^30^, and several letters to the editor in response to previous publications on oesophageal candidiasis.^31^,^32^

So relax and enjoy this exotic menu of papers in *African Health Sciences*.

James K Tumwine Editor in Chief, *African Health Sciences*.
